# Erythrocyte Inosine triphosphatase activity: A potential biomarker for adverse events during combination antiretroviral therapy for HIV

**DOI:** 10.1371/journal.pone.0191069

**Published:** 2018-01-12

**Authors:** N. Chantal Peltenburg, Jörgen Bierau, Jaap A. Bakker, Jolanda A. Schippers, Selwyn H. Lowe, Aimée D. C. Paulussen, Bianca J. C. van den Bosch, Mathie P. G. Leers, Bettina E. Hansen, Annelies Verbon

**Affiliations:** 1 Department of Internal medicine, Division Infectious Diseases, Erasmus Medical Center, Rotterdam, The Netherlands; 2 Department of Medical Microbiology and Infectious Diseases, Erasmus Medical Center, Rotterdam, The Netherlands; 3 Department of Clinical Genetics, Maastricht University Medical Center, Maastricht, The Netherlands; 4 Department of Clinical Chemistry and Laboratory Medicine, Leiden University Medical Center, Leiden, The Netherlands; 5 Department of Integrated Care, Maastricht University Medical Center, Maastricht, The Netherlands; 6 Department of internal medicine, Division Infectious Diseases, Maastricht University Medical Center, Maastricht, The Netherlands; 7 Department of Medical Microbiology, School of CAPHRI, Maastricht University Medical Center, Maastricht, The Netherlands; 8 Department of Clinical Chemistry & Hematology, Zuyderland Medical Center, Heerlen, The Netherlands; 9 Department of Gastroenterology & Hepatology, Erasmus Medical Center, Rotterdam, The Netherlands; Universidad Autonoma de Madrid Centro de Biologia Molecular Severo Ochoa, SPAIN

## Abstract

The purine analogues tenofovir and abacavir are precursors of potential substrates for the enzyme Inosine 5’-triphosphate pyrophosphohydrolase (ITPase). Here, we investigated the association of ITPase activity and *ITPA* genotype with the occurrence of adverse events (AEs) during combination antiretroviral therapy (cART) for human immunodeficiency virus (HIV) infection. In 393 adult HIV-seropositive patients, AEs were defined as events that led to stop of cART regimen. ITPase activity ≥4 mmol IMP/mmol Hb/hour was considered as normal. *ITPA* genotype was determined by testing two *ITPA* polymorphisms: c.94C>A (p.Pro32Thr, rs1127354) and c.124+21A>C (rs7270101). Logistic regression analysis determined odds ratios for developing AEs. In tenofovir-containing regimens decreased ITPase activity was associated with less AEs (p = 0.01) and longer regimen duration (p = 0.001). In contrast, in abacavir-containing regimens decreased ITPase activity was associated with more AEs (crude p = 0.02) and increased switching of medication due to AEs (p = 0.03). *ITPA* genotype wt/wt was significantly associated with an increase in the occurrence of AEs in tenofovir-containing regimens. Decreased ITPase activity seems to be protective against occurrence of AEs in tenofovir-containing cART, while it is associated with an increase in AEs in abacavir-containing regimens.

## Introduction

Combination anti-retroviral therapy (cART) for patients infected with the human immunodeficiency virus (HIV) has been increasingly effective over the last years. However, adverse events (AEs) are still common and can be severe. Predicting whether AEs will occur with specific antiretroviral drugs would be a valuable tool in the choice of cART regimens. Although determination of HLA-B*57:01 status to predict hypersensitivity for abacavir, is widely used [1], no other biomarkers or genetic susceptibility traits are known that can be used to predict AEs associated with cART.

The enzyme Inosine 5’-triphosphate pyrophosphohydrolase (ITPase) is an enzyme which converts Inosine 5’-triphosphate (ITP) to Inosine 5’-monophosphate (IMP) and Xanthosine 5’-triphosphate (XTP) to Xanthosine 5’-monophosphate (XMP). IMP and XMP are central metabolites in the purine metabolism, from which Adenosine 5’-monophosphate (AMP) and Guanosine 5’-monophosphate (GMP) and subsequently Adenosine 5’-triphosphate (ATP) and Guanosine 5’-triphosphate (GTP) can be formed. ITPase is encoded by the *ITPA* gene located on chromosome 20p. *ITPA* is a polymorphic gene and a substantial part of the Western population carries one of the single nucleotide polymorphisms (SNPs) *ITPA* c.94C>A or *ITPA* c.124+21A>C [2]. The frequency of c.94C>A is highest in Asian populations (14–10%) compared to Central/South American (1–2%) and Caucasian and African populations (6–7%) [2]. The SNP c.124+21A>C is extremely rare in Asian populations [3–5]. These polymorphisms result in a decreased ITPase activity [6, 7]. Homozygosity for *ITPA* c.94 C>A leads to a null activity solely in erythrocytes, while activity in nucleated cells remains detectable [8]. True ITPase deficiency in humans is very rare and causes a severe form of early infantile encephalopathy [9]. Decreased ITPase activity and the frequent *ITPA* SNPs are associated with a reduced risk to develop ribavirin-induced haemolytic anaemia in patients on treatment for hepatitis C [10–12], and with an increased risk of AEs in patients treated with thiopurines [13–15].

Our interest in the role of ITPase in the treatment of HIV-infected patients stems from the fact that both abacavir and tenofovir as well as didanosine are purine analogues and are therefore potential precursors of substrates for ITPase. Similarly to its association with AEs during the use of thiopurines and ribavirin, ITPase activity might influence the occurrence of AEs during purine analogues containing cART. Moreover, the majority of HIV-infected patients showed a decreased erythrocyte ITPase activity compared to healthy controls [16]. Therefore, we determined whether ITPase activity and *ITPA* genotype are associated with the occurrence of AEs during cART with purine analogues in a cohort of HIV-infected patients. In addition, we tested whether the active metabolites of abacavir and tenofovir (i.e. carbovir-triphosphate and tenofovir-diphosphate resp.) are substrates for the enzyme ITPase.

## Materials and methods

### Patients

Consecutive HIV-infected patients attending the outpatient clinic of the Maastricht University Medical Center in Maastricht, The Netherlands, between March first 2009 and January first 2014, who were treated with cART were included in this study after providing a written informed consent. Demographic, laboratory and clinical data from the moment the patients entered medical care for the HIV-infection until January first 2014 were retrieved from the database of the Dutch HIV monitoring foundation (Stichting HIV Monitoring; SHM), also known as the AIDS Therapy Evaluation in the Netherlands (ATHENA) and if needed from the medical records. The SHM includes data on demographics, comorbidities, cART, clinical, immunological and virological parameters of individuals in HIV care since January 1996 in any of the 26 HIV treatment centers in the Netherlands. Patients can opt-out after being informed by their treating physician of the purpose of collection of clinical data. The study was censored at January first 2014.

The study was performed according to the Helsinki Declaration and approved by the Medical Ethical Committee of the Maastricht University Medical Center, Maastricht, The Netherlands.

### ITPase activity

Erythrocyte ITPase activity was determined once per patient in the period of March first 2009 until January first 2014 and determined as described previously [17] and assessed by formation of inosine monophosphate (IMP) from inosine triphosphate (ITP). ITPase activity was expressed as mmoles of IMP formed from ITP in one hour per mmol hemoglobin (mmol IMP/mmol Hb/hour). The cut-off value discriminating between normal or decreased ITPase activity was set at 4 mmol IMP/mmol Hb/hour, which is the lowest value within the 95% CI for *ITPA* wt/wt carriers [16, 18].

In order to test whether or not carbovir-triphosphate and tenofovir-diphosphate are substrates for ITPase, the enzyme activity assay was carried out as described, using 1 millimolar of ITP, carbovir-triphosphate or tenofovir-diphosphate. Erythrocytes of a non-HIV infected confirmed wild type *ITPA* genotype carrier individual was used for this experiment. Experiments were performed in triplicate.

Carbovir-triphosphate and tenofovir-diphosphate were obtained from Toronto Research Chemicals (Toronto, Ontario, Canada).

### *ITPA* genotype analysis

Genomic DNA was isolated from whole blood using the Wizard Genomic DNA purification kit (Promega, Madison, WI) and genotyped using sanger sequencing for the two *ITPA* polymorphisms; c.94C>A (p.Pro32Thr, rs1127354) and c.124+21A>C (rs7270101), as previously described [16]. When both polymorphisms were not detected, the genotype was considered to be wild type (wt/wt). All sequences were evaluated by two independent laboratory experts.

### Adverse events

For a uniform definition of AEs, AEs resulting in stopping or switching of the cART regimen and AEs that could be objectified in the laboratory were included. AEs were defined as stopping or switching for any reason except for the reasons virological failure, interaction with other medication, simplification or intensification of the regimen, drug taken off the market, patient deceased, low cART blood levels. Before statistical analyses, AEs were categorised and their potential association with ITPase activity or *ITPA* genotype was determined. The categorisation of the AEs was as follows: any AE (all categories of AE named hereafter taken together), gastro-intestinal (abdominal pain, nausea, diarrhoea, stomach ache, loss of appetite, pancreatitis), neurological (psychiatric complaints or dizziness, sleeping disorder, headache, tremor, disorders of taste), renal (renal insufficiency, kidney stones, nephritis, hypophosphatemia or lactate acidosis as reported reason for stopping the cART regimen or MDRD <60 ml/min/1.73 m^2^ or phosphate <0.6 mmol/L in at least two separate measurements without other obvious cause), skin (rash or abscess at the site of injection) and liver related (liver failure as reported reason for stopping the cART regimen or alanine aminotransferase and/or aspartate aminotransferase >90 U/L without other obvious cause, in at least two separate measurements, or in one measurement in case of only two measurements performed during that regimen).

### Statistical analysis

Results were analysed using IBM SPSS Statistics 21 (IBM Corporation, New York, USA) and SAS system for windows version 9.3. Pearson-chi-square-tests, Fisher’s exact test and independent samples T-tests were used to determine significant differences. *P* values <0.05 were considered to be statistically significant. The occurrence of adverse events was analyzed with logistic regression with repeated statement and adjusted for cumulative total duration of cART therapy, cumulative duration of purine analogue therapy of all prior regimens and duration of the current regimen. Analysis included check for significant interaction with treatment and ITPase activity. When abacavir, tenofovir or didanosine were used concomitantly in one cART regimen, these regimens were excluded from the analysis.

## Results

### Patient characteristics

Of 393 HIV infected patients, 205 (52.2%) patients had a decreased ITPase activity (Table 1). There were no statistically significant differences with respect to age, gender, ethnicity and alcohol use between the groups having normal and decreased ITPase activity. Mean CD4 nadir did not differ significantly between the group of patients with decreased ITPase activity (216 ± 161 cells/μL) and normal ITPase activity (200 ± 146 cells/μL).

**Table 1:** Demographic and clinical characteristics of the patients (n = 393) with ITPase activity <4 and ≥4 mmol IMP/mmol Hb/hour.

Characteristic	ITPase activity <4^a^ (n = 205)	ITPase activity ≥4^a^ (n = 188)	P-value
**Age**; median (min-max) (years)	50.6 (20–81)	49.7 (27–84)	0.80
**Male Gender** (n / %)	164 (80.0)	155 (82.4)	0.53
**Race** (n / %)			0.81
White non-Hispanic	164 (80.0)	147 (78.2)	
White Hispanic	5 (2.4)	4 (2.1)	
Black	22 (10.7)	27 (14.4)	
Asian or other	14 (6.9)	10 (5.3)	
**ITPase activity** (mean±S.D.)	2.44 (1.12)	5.24 (1.09)	<0.001
***ITPA* genotype** (n / %)			<0.001
Wt/wt	90 (43.9)	175 (93.1)	
Wt/c.124+21A>C	59 (28.8)	9 (4.8)	
Wt/c.94C>A or other^b^	53 (25.9)	-	
Unknown	3 (1.5)	4 (2.1)	
**Alcohol** (IU/day)			0.51
<2	157 (76.6)	133 (70.7)	
≥2	35 (17.1)	36 (19.1)	
Unknown	13 (6.3)	19 (10.1)	
**Recreational drugs**^c^			0.04
None	120 (58.5)	92 (48.9)	
Yes	50 (24.4)	55 (29.3)	
Unknown	35 (17.1)	41 (21.8)	
**Hepatitis B co-infection**			0.25
No / cleared	182 (88.8)	167 (88.8)	
Yes	10 (4.9)	6 (3.2)	
Unknown	13 (6.3)	15 (8.0)	
**Hepatitis C co-infection**			0.52
No	174 (84.9)	159 (84.6)	
Yes	27 (13.2)	22 (11.8)	
Unknown	4 (2.0)	7 (3.7)	
**CD4 nadir** (mean±S.D.)	215.6 ± 160.9	200.1 ± 145.7	0.32
**Year of start** (median / min-max)	2006 (1987–2013)^d^	2006 (1987–2013)^e^	0.25
**% of patients starting cART before the year 1998**	9.7	8.3	0.68
**Number of cART regimens per patient** (median / min-max)	3 (1–14)	3 (1–18)	0.74
**Total number of cART regimens**	734	688	
**Duration of cART regimen in months** (median / min-max)	18.0 (0–161)^f^	15.5 (0–160)^g^	0.06
**Purine containing cART (n / %)** Tenofovir Abacavir Didanosine	306 (40.1) 131 (17.2) 51 (6.7)	295 (41.1) 113 (15.8) 77 (10.7)	0.09
**Duration purine containing cART in months (**mean±S.D.) Tenofovir Abacavir Didanosine	29.2 ± 24.7 34.9 ± 35.6 24.7 ± 27.9	22.5 ± 22.0 41.4 ± 40.8 19.4 ± 22.4	0.001 0.19 0.24

^a^ mmol IMP/mmol Hb/hour

^b^ Other = homozygous c.124+21A>C or homozygous c.94C>A or heterozygous c.124+21A>C/c.94C>A

^c^ heroin, cocaine, amphetamines, 3,4-methylenedioxymethamphetamine (MDMA), cannabis, lysergic acid diethylamide (LSD), ketamine, gamma-hydroxybutyric acid (GHB) and akylnitrate (‘poppers’)

^d^ Year of start missing in 1 patient

^e^ Year of start missing in 2 patients

^f^ Duration of cART regimen missing in 1 patient

^g^ Duration of cART regimen missing in 3 patients

### cART regimens

In total 393 patients accounted for 1464 prescribed regimens. The median number of regimens per patient was 3, with a maximum of 18 regimens in one patient (Table 1). In total 38.992 person months of anti-retroviral therapy were observed. 9% of the regimens started before 1998. Purine analogues (tenofovir, abacavir and didanosine) were frequently prescribed (n = 601, n = 244 and n = 128 respectively) and the proportion of purine analogue containing regimens (69.3%) did not differ between the group of patients with normal ITPase activity and the group of patients with decreased ITPase activity (p = 0.09). 17 regimens (1.1%) contained both tenofovir and didanosine, 16 regimens (1.1%) contained abacavir and didanosine and 9 regimens (0.6%) contained tenofovir and abacavir. These regimens were excluded from further analysis, so 1422 regimens were used to assess the association of ITPase activity and *ITPA* genotype with AEs.

### ITPase activity and *ITPA* genotype in HIV patients

*ITPA* genotype was determined in 386 patients. The most prominent *ITPA* genotype was wt/wt (265 patients, 67.4%). The occurrence of wt/c.124+21A>C and wt/c.94C>A *ITPA* genotype variants was 68 (17.3%) and 35 (8.9%) respectively. Other variants occurred in only 4.6% of the patients (homozygous c.124+21A>C n = 5, homozygous c.94C>A n = 2 and heterozygous c.124+21A>C/c.94C>A n = 11). Mean ITPase activity correlated with *ITPA* genotype, with the highest ITPase activity in the wt/wt genotype. However, within the *ITPA* genotype wt/wt, 90 (34.0%) patients had decreased ITPase activity and within the *ITPA* genotype wt/c.124+21A>C, 9 (13.2%) of the patients had normal ITPase activity. The other *ITPA* genotypes were only associated with a decreased ITPase activity.

### Association of ITPase activity with AEs

**Fig 1:**
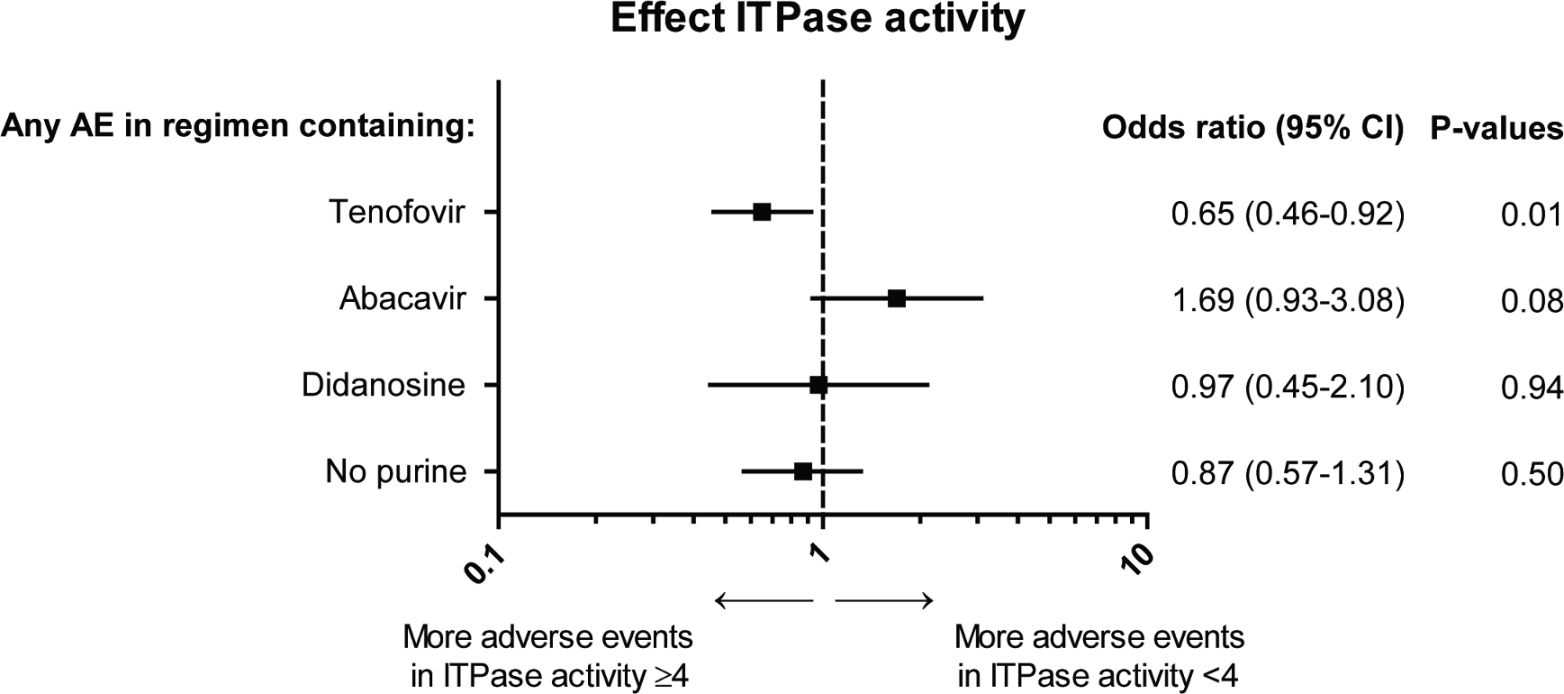
Effect ITPase activity on total adverse events. The effect of decreased versus normal ITPase activity on the occurrence of total adverse events (grouped by regimens containing tenofovir, abacavir or didanosine and regimens without tenofovir, abacavir or didanosine) is plotted. Odds ratio with 95% confidential interval and matching p-values are displayed. The analyses were conducted with repeated statement, adjusted for cumulative total duration of cART, cumulative duration of purine analogue therapy of all prior regimens and duration of the current regimen.

734 regimens were prescribed in 205 patients with decreased ITPase activity and 688 in 188 patients with normal ITPase activity (N.S., Table 1). In 6.8% of the regimens the reason for switching or stopping cART regimen was unknown. In total, AEs were present during 714 regimens (50.2%) with 356 AEs in patients with decreased ITPase activity and 358 in patients with normal ITPase activity (N.S.). The occurrence of AEs and the effect of the ITPase activity are displayed in Table 2 and Fig 1 respectively.

**Table 2:** Occurrence of adverse events in patients grouped by ITPase activity, percentage of adverse events and crude p-values per purine type.

cART regimen	ITPase activity <4^a^		ITPase activity ≥4^a^		Crude p-value
	Adverse events (n) / Patients (n)	% of total AE	Adverse events (n) / Patients (n)	% of total AE	
Tenofovir	137/306	45.1%	167/295	54.9%	0.004
Abacavir	74/131	61.2%	47/113	38.8%	0.02
Didanosine	27/51	40.3%	40/77	59.7%	0.91
No purine	118/246	53.2%	104/203	46.8%	0.49

^a^ mmol IMP/mmol Hb/hour

#### Tenofovir

Normal ITPase activity was significantly associated with AEs in regimens containing tenofovir (167 vs 137 respectively, Odds ratio (OR) 0.65 for decreased ITPase activity versus normal activity; 95% CI 0.46–0.92; p = 0.01), see Fig 1. Tenofovir-containing regimens used with normal ITPase activity were significantly more often switched because of AEs than for other reasons compared to regimens used with a decreased ITPase activity (91 of 281 vs 70 of 293 respectively (reason for switch unknown in 27 regimens), p = 0.02). Furthermore, mean regimen duration was statistically significantly longer in patients with a decreased ITPase activity (29.2 vs 22.5 months; p = 0.001), see Table 1.

**Fig 2:**
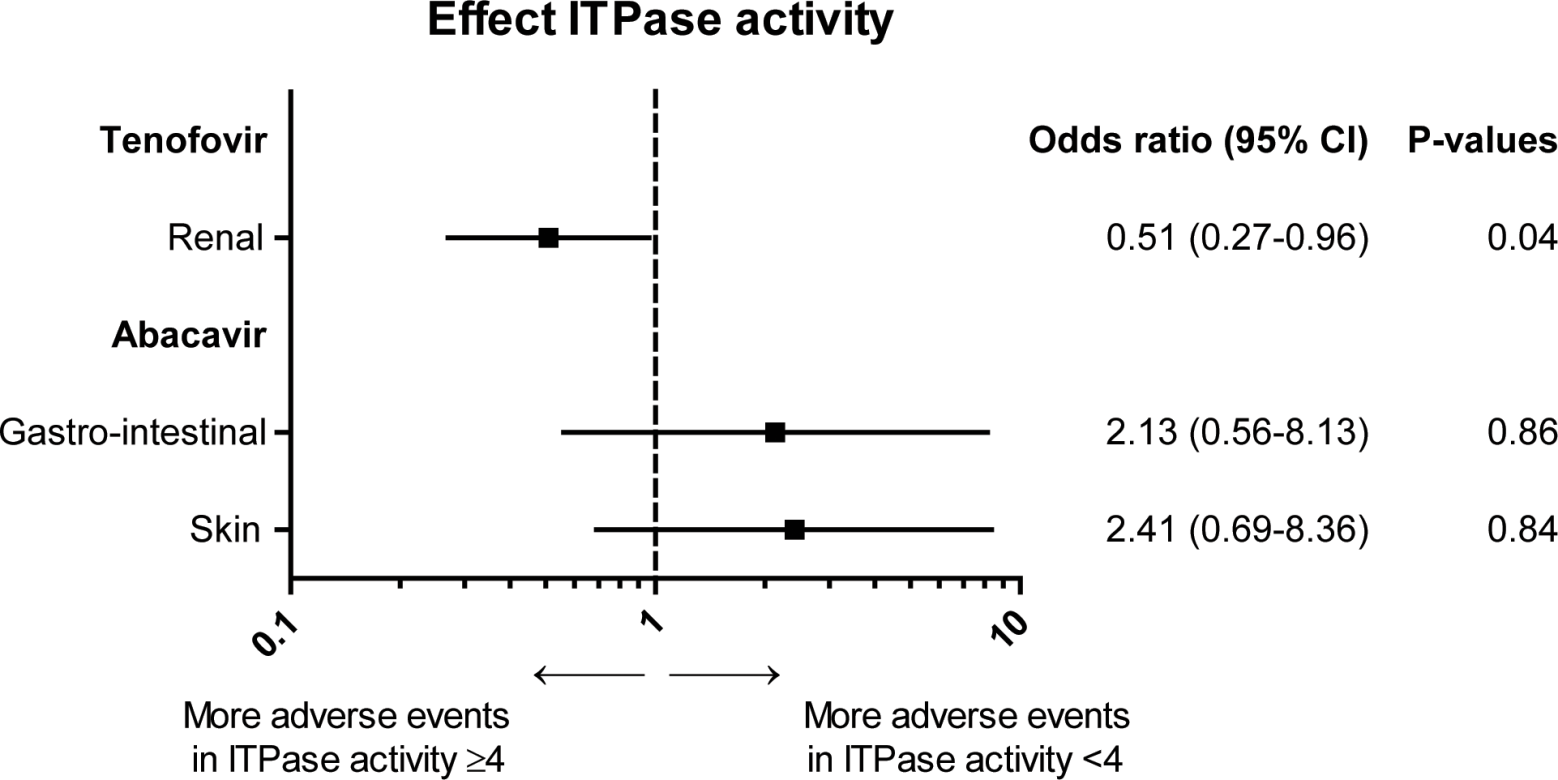
Effect ITPase activity on regimen associated adverse events. The effect of decreased ITPase activity on the occurrence of regimen associated adverse events (renal adverse events for tenofovir and gastro-intestinal and skin related adverse events for abacavir) is plotted. Odds ratio with 95% confidential interval and matching p-values are displayed. The analyses were conducted with repeated statement, adjusted for cumulative total duration of cART, cumulative duration of purine analogue therapy of all prior regimens and duration of the current regimen.

Of all the renal AEs that occurred, 48.7% were in the group of regimens containing tenofovir (n = 55) and of these 63.6% occurred in the patients with normal ITPase activity (p = 0.04; OR 0.51; 95% CI 0.27–0.96 for patients with decreased ITPase activity versus normal activity), see Fig 2.

#### Abacavir

In regimens containing abacavir 61.2% of all AEs occurred in the patients with decreased ITPase activity and 38.8% in the patients with normal ITPase activity (crude p = 0.02). After correction using logistic regression, OR remained elevated for decreased ITPase activity versus normal ITPase activity (1.69) but did not reach significance (p = 0.08), see Fig 1. Significantly more often the cART regimen was switched because of AEs instead of other reasons when ITPase activity was decreased compared to normal ITPase activity (50 of 124 vs 29 of 108 respectively (reason for switch unknown in 12 regimens), p = 0.03). In general, more adverse events tended to occur in patients with decreased ITPase activity, e.g. gastrointestinal and skin related AEs (Fig 2). Of the cART regimens containing abacavir, 6.1% were stopped or changed because of skin related AEs and 73.3% of these events occurred in the patients with decreased ITPase activity, however this difference was not statistically significant.

#### Didanosine and regimens without tenofovir, abacavir and didanosine

No statistically significant association was found for AEs occurring with use of didanosine or regimens without tenofovir, abacavir and didanosine and ITPase activity.

### Association of *ITPA* genotype with AEs

**Fig 3:**
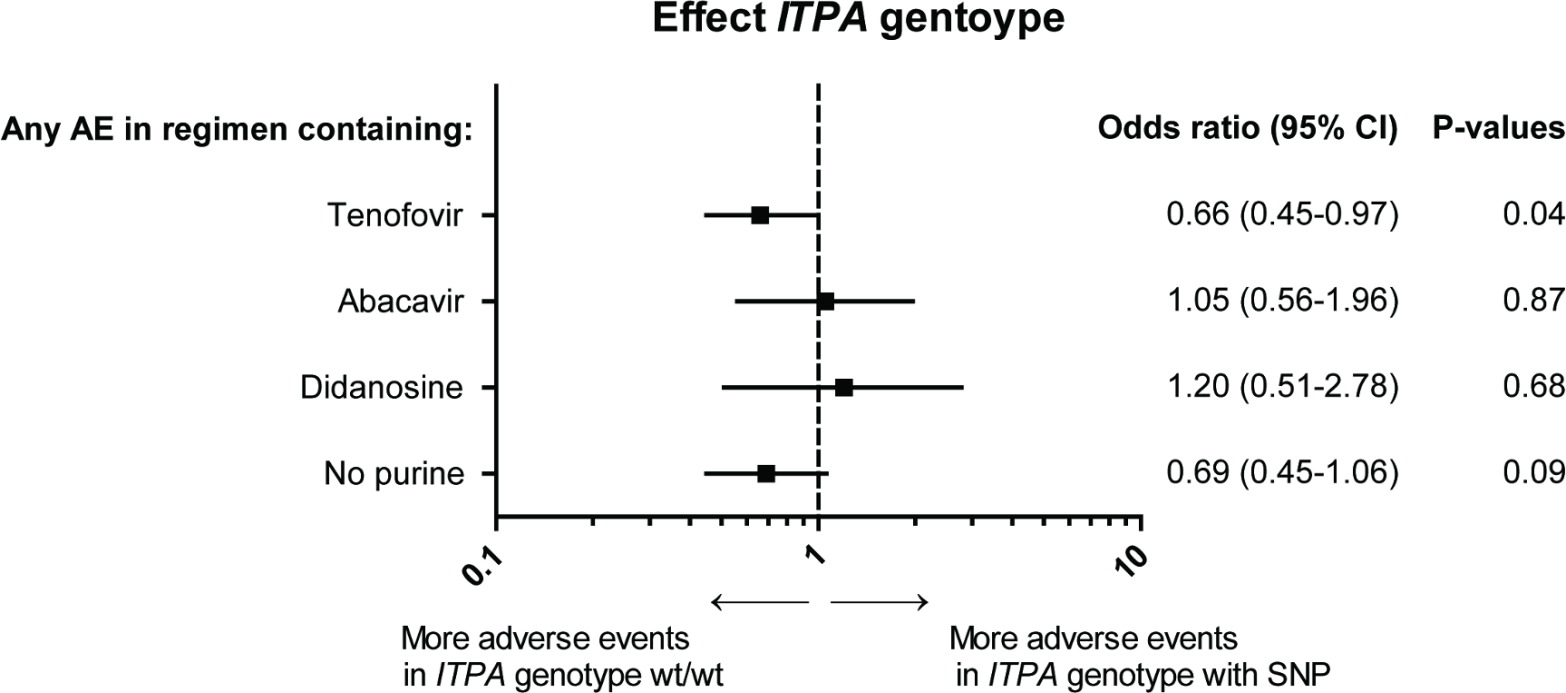
Effect *ITPA* genotype on total adverse events. The effect of *ITPA* genotype wt/wt versus the other *ITPA* genotypes on the occurrence of total adverse events (grouped by regimens containing tenofovir, abacavir or didanosine and regimens without tenofovir, abacavir or didanosine) is plotted. Other *ITPA* genotypes than wt/wt were wt/c.124+21A>C, wt/c.94C>A, c.124+21A>C/c.124+21A>C, c.94C>A/c.94C>A or c.124+21a>C/c.94C>C. Odds ratio with 95% confidential interval and matching p-values are displayed. The analyses were conducted with repeated statement, adjusted for cumulative total duration of cART, cumulative duration of purine analogue therapy of all prior regimens and duration of the current regimen.

The occurrence of AEs and the effect of the *ITPA* genotype are displayed in Table 3 and Fig 3 respectively. 960 regimens were prescribed for patients with wt/wt genotype and 435 for patients with one or more of the SNPs. Genotype was unknown for 27 regimens.

**Table 3:** Occurrence of adverse events in patients grouped by *ITPA* genotype, and crude p-values per purine type for each type of adverse event.

cART regimen	SNP in *ITPA* genotype^a^		*ITPA* genotype wt/wt		Genotype unknown		Crude p-value
	Adverse events (n) / Patients (n)	% of total AE	Adverse events (n) / Patients (n)	% of total AE	Adverse events (n) / Patients (n)	% of total AE	
Tenofovir	80/186	26.3%	220/409	72.4%	4/6	1.3%	0.04
Abacavir	38/75	31.4%	82/164	67.8%	1/5	0.8%	0.52
Didanosine	18/31	26.9%	48/96	71.6%	1/1	1.5%	0.54
No purine	64/143	28.8%	151/291	68.0%	7/15	3.2%	0.36

^a^ heterozygous wt/c.124+21A>C or wt/c.94C>A or homozygous c.124+21A>C or homozygous c.94C>A or compound heterozygous c.124+21A>C/c.94C>A

#### Tenofovir

**Fig 4:**
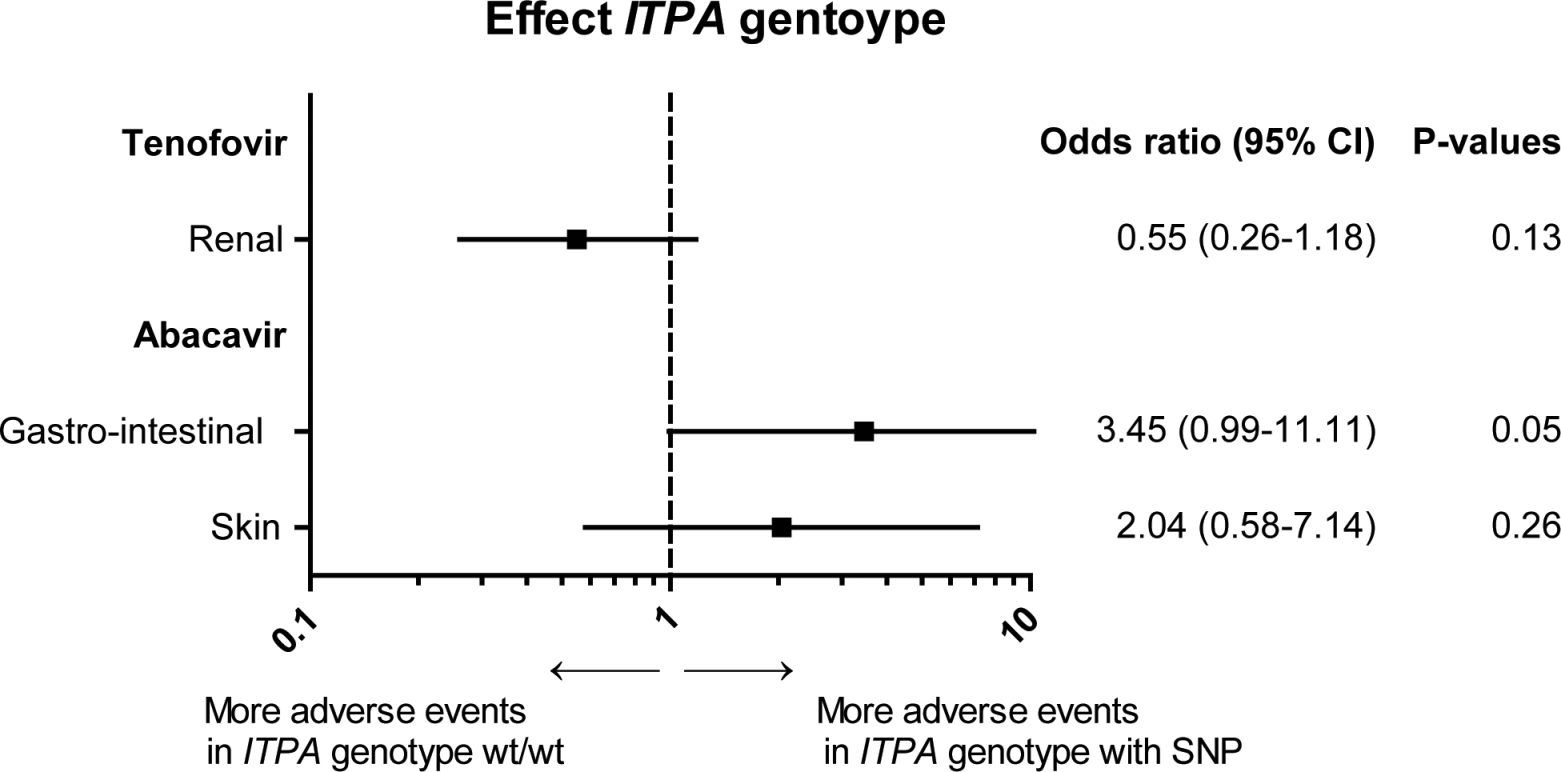
Effect *ITPA* genotype on regimen associated adverse events. The effect of *ITPA* genotype wt/wt versus the other *ITPA* genotypes on the occurrence of regimen associated adverse events (renal adverse events for tenofovir and gastro-intestinal and skin related adverse events for abacavir) is plotted. Other *ITPA* genotypes than wt/wt were wt/c.124+21A>C, wt/c.94C>A, c.124+21A>C/c.124+21A>C, c.94C>A/c.94C>A or c.124+21a>C/c.94C>C. Odds ratio with 95% confidential interval and matching p-values are displayed. The analyses were conducted with repeated statement, adjusted for cumulative total duration of cART, cumulative duration of purine analogue therapy of all prior regimens and duration of the current regimen.

*ITPA* genotypes other than wt/wt, associated with decreased ITPase activity, seemed to be protective against AEs (p = 0.04), as 72.4% of all AEs occurred in the patients with *ITPA* genotype wt/wt and 26.3% in patients with SNPs in the genotype. 1.3% Of the regimens with an AE had an unknown genotype. 76.4% of all renal AEs occurred in the regimens with wt/wt genotype, this difference was not statistically significant, see Fig 4.

#### Abacavir

No association was found between *ITPA* genotype and all AEs in patients using abacavir. For gastro-intestinal AEs a SNP in the *ITPA* genotype was associated with an increased number of AEs (OR 3.45 for *ITPA* genotypes with SNPs versus wt/wt genotype; 95% CI 0.99–11.11; p = 0.05), see Fig 4.

#### Didanosine and regimens without tenofovir, abacavir and didanosine

No association was found between *ITPA* genotype and AEs in patients using didanosine or regimens without tenofovir, abacavir and didanosine.

### Carbovir-triphosphate and tenofovir-diphosphate are no direct substrates for ITPase

To test the hypothesis that carbovir-triphosphate could be a substrate for ITPase, resulting in higher carbovir levels when ITPase activity is decreased, and more pronounced adverse effects, carbovir-triphosphate was directly used as a substrate for ITPase. Carbovir-triphosphate was not a direct substrate for ITPase and neither was tenofovir-diphosphate.

## Discussion

Here, we show for the first time that ITPase activity is associated with the occurrence of AEs in patients using cART containing the purine analogues tenofovir and abacavir. A significant reduction in all AEs was found in patients with decreased ITPase activity using tenofovir. Moreover, mean regimen duration was significantly longer indicating a better tolerance of tenofovir containing cART regimens in patients with decreased ITPase activity. On the other hand, patients with decreased ITPase activity using abacavir were more at risk for developing AEs. Mean regimen duration was longer in the patients with normal ITPase activity, indicating a better tolerance of abacavir in these patients. No clear association between AEs and regimens without tenofovir, abacavir or didanosine could be demonstrated.

**Fig 5:**
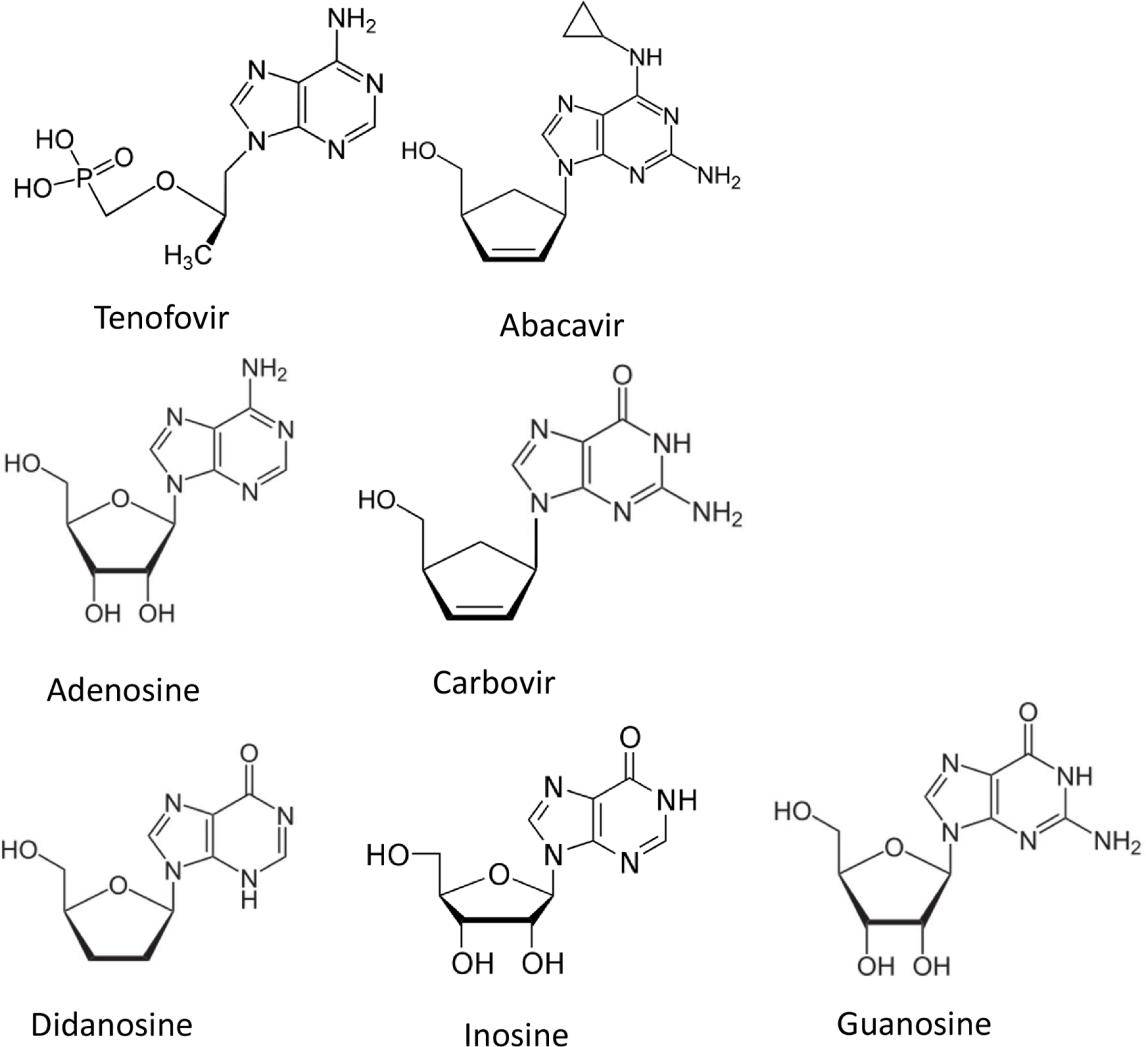
Chemical structures. Chemical structures of tenofovir, abacavir, adenosine, carbovir, didanosine, inosine and guanosine.

These data suggest that ITPase activity may be used as a pharmacogenetic biomarker in patients starting cART containing tenofovir or abacavir. Up to now no other biomarkers are in use, apart from HLA-B*57:01 status to predict abacavir hypersensitivity syndrome. As tenofovir is used as a part of the regimen in the PrEP studies to prevent HIV transmission, we expect an increase in the use of tenofovir by indivuduals not infected with HIV, A test to identify individuals with increased risk of developing long term adverse effects due to tenofovir would be an extremely welcome asset. The present study suggest ITPase activity is a potential candidate.In other diseases, results of studies using *ITPA* polymorphisms to predict AEs varied with the purine analogue used. The ITPase lowering *ITPA* polymorphisms were shown to be protective against ribavirin-induced anaemia in hepatitis C on treatment [19, 20]. In patients with inflammatory bowel disease using azathioprine, however, adverse events occurred more frequently in patients with ITPase lowering *ITPA* polymorphisms [7, 21]. In our study a lower ITPase activity was associated with less cART regimen switches due to AEs in patients using tenofovir, whereas regimens containing abacavir were more frequently switched. The underlying cause of the observed differences between the purine analogues tenofovir and abacavir is yet unclear. Both abacavir and tenofovir are potent inhibitors of HIV reverse transcriptase [22–24]. However, the chemical structure differs greatly between the two analogues (Fig 5). Whilst tenofovir is an acyclic adenine nucleotide analogue, abacavir is the prodrug of carbovir, which is formed by removal of the cyclopropylammonia moiety attached to the purine base and is a guanosine analogue.

We expect that part of the explanation is to be found in the chemistry of these compounds.

In our hands, both carbovir-triphosphate and tenofovir-diphosphate proved to be non-substrates for ITPase, so the mechanism behind our observation that decreased ITPase activity leads to an increased occurrence of adverse events in abacavir therapy is not easily explained. As was hypothesized by Coelho et al [25], the use of cART may lead to an increase in IMP and so to an increase in cytotoxicity by an increased ITP level in patients with decreased ITPase activity. Interestingly, we observed cultured skin fibroblasts of patients with encephalopathy associated with *ITPA* mutations [9] leading to severely reduced ITPase activity show increased levels of IMP rather than ITP when compared to controls, so direct toxicity by ITP can only explain part of the mechanism involved. Bondoc and coworkers [26] demonstrated that intracellular anabolism of carbovir was stimulated by adenine, adenosine, hypoxanthine, inosine and even dideoxy-Inosine. Their hypothesis is that increased intracellular levels of IMP and ATP enhanced the anabolism of carbovir by stimulating phosphorylation by 5’-nucleotidase. In line with this, we might hypothesize that a decreased ITPase activity leads to an increase in IMP and so to an increase of carbovir anabolism. In addition to its anti-retroviral activity, carbovir-triphosphate has been demonstrated to be a competitive inhibitor of soluble guanylate cyclase influencing platelet activity [27]. Considering that carbovir-triphosphate might be increased by decreased ITPase activity, the interference of carbovir-triphosphate in the nitric oxide (NO) signalling pathway may be considerable, leading to adverse events and regimen change. This hypothesis needs further investigation to clarify the mechanism.

Skin reactions related to abacavir are an immunological phenomenon. In our study, most of the skin related adverse events occurred in the abacavir-containing regimens used by patients with decreased ITPase actvitiy, although this difference was not statistically significant. The role of ITPase in immunologically induced AEs remains to be elucidated.

For tenofovir an explanation for the present results seems to be less straightforward. Tenofovir differs from carbovir in two essential aspects: tenofovir is a nucleoside-monophosphate and a adenosine analogue rather than a guanosine analogue. Tenofovir metabolites appear to be resistant to deamination [28] making accumulation of the deaminated tenofovir-diphosphate metabolite due to decreased ITPase activity unlikely. At this moment we can only speculate that ITPase activity influences tenofovir metabolism in a yet unknown fashion. In this study we were not able to rule out other factors that may have influenced renal events other than tenofovir use, like hypertension, diabetes mellitus, age and the use of other medication, like non-steroidal anti-inflammatory drugs.

All previous studies evaluating the effect of *ITPA* genotype polymorphisms on adverse events assumed that the reported ITPase activity directly corresponds to a specific *ITPA* polymorphism [14, 18]. However, in accordance with our previous work [29] we found that an *ITPA* variant such as wt/c.124+21A>C lead to a variety of ITPase activities from as low as 1.53 to as high as 7.70 mmol IMP/mmol Hb/hour. Moreover, in spite of wt/wt genotype, HIV-infected patients were found to have a decreased ITPase activity compared to control patients [16] and therefore more often will have an ITPase activity <4 mmol IMP/mmol Hb/hour. In HIV, the association between *ITPA* genotype and ITPase activity is less strict as has previously been assumed. This may be an explanation why *ITPA* genotype correlated less to AEs compared to ITPase activity, as we found in this study. Previously we showed that ITPase activity is lower in HIV-infected patients compared to control populations in erythrocytes [16] as well as in leukocytes [30] independent of *ITPA* genotype, which did not appear to be an effect of nucleoside analogues [16].

Some limitations of this research need to be mentioned. Because cART is, by definition, a combination of antiretroviral drugs, adverse events during a cART regimen might be attributed to more than one drug. However, by using repeated statement and adjusting the statistical analysis for cumulative total duration of cART, cumulative duration of purine analogue therapy of all prior regimens and duration of the current regimen, we were able to measure the association between ITPase activity and tenofovir and abacavir containing regimens. More studies will be needed to confirm our findings.

The lack of more significant results analyzing specific adverse events other than gastro-intestinal, renal and skin related adverse events in this study may be due to the relatively small numbers of the occurrence of these adverse events. This was probably due to the fact that in our definition of adverse event we only used the reasons for stopping a regimen in combination with retraceable laboratory values. We have chosen this strategy because in the SHM database reason for switching cART regimen is a mandatory question, whereas reporting AEs is up to the physician and not a prerequisite item in the database and therefore is a less reliable parameter. Still, in 7% of the regimens, the reason for switching cART was unknown. The number of regimens with unknown reason for switching were equally distributed between both ITPase activity groups. We therefore do not expect this to be affecting the results of the study.

Because the data were collected retrospectively, the causality between the use of cART and the occurrence of AEs cannot be proven in this study. By using only the reasons for stopping the cART regimen or retraceable laboratory values, we avoided some bias, however, prospective trials are needed to confirm our data.

In conclusion, ITPase enzyme activity <4 mmol IMP/mmol Hb/hour seems to be protective against occurrence of adverse events in cART regimens containing tenofovir, while it leads to an increase in adverse events in cART regimens containing abacavir. These results warrant further elucidation of the mechanism involved and need to be confirmed in a prospective trial.

## Abbreviations

cART: combination anti-retroviral treatment
HIV: human immunodeficiency virus
AEs: Adverse events
ITPase: Inosine 5’-triphosphate pyrophosphohydrolase
ITP: Inosine 5’-triphosphate
IMP: Inosine 5’-monophosphate
XTP: Xanthosine 5’-triphosphate
XMP: Xanthosine 5’-monophosphate
AMP: Adenosine 5’-monophosphate
GMP: Guanosine 5’-monophosphate
ATP: Adenosine 5’-triphosphate
GTP: Guanosine 5’-triphosphate
SNPs: Single-nucleotide polymorphisms
SHM: Stichting HIV monitoring (Dutch association of HIV monitoring)
Hb: Hemoglobin
OR: Odds ratio
CI: Confidence interval
NO: nitric oxide
